# Altered resting state functional connectivity of anterior cingulate cortex in drug naïve adolescents at the earliest stages of anorexia nervosa

**DOI:** 10.1038/srep10818

**Published:** 2015-06-04

**Authors:** Santino Gaudio, Claudia Piervincenzi, Bruno Beomonte Zobel, Francesca Romana Montecchi, Giuseppe Riva, Filippo Carducci, Carlo Cosimo Quattrocchi

**Affiliations:** 1 Departmental Faculty of Medicine and Surgery, Università “Campus Bio-Medico di Roma”, Rome, Italy; 2 Eating Disorders Centre “La Cura del Girasole” ONLUS, Rome, Italy; 3 Department of Physiology and Pharmacology, Neuroimaging Laboratory, Sapienza University, Rome, Italy; 4 Institute for Advanced Biomedical Technologies, University of G. d’Annunzio Chieti-Pescara, Chieti, Italy; 5 Applied Technology for Neuro-Psychology Lab, Istituto Auxologico Italiano, Milan, Italy; 6 Department of Psychology, Università Cattolica del Sacro Cuore, Milan, Italy

## Abstract

Previous Resting-State Functional Connectivity (RSFC) studies have shown several functional alterations in adults with or recovered from long Anorexia Nervosa (AN). The aim of this paper was to investigate whole brain RSFC in adolescents with AN in the earliest stages, less than 6 months, of the disorder. Sixteen drug-naïve outpatient female adolescents with AN-restrictive type (AN-r) (mean age: 15,8; SD 1,7) were compared to 16 age-matched healthy female (mean age: 16,3; SD 1,4). Relevant resting state networks (RSNs) were identified using independent component analysis (ICA) from functional magnetic resonance imaging data; a dual regression technique was used to detect between-group differences in the RSNs. Between-group differences of the functional connectivity maps were found in the executive control network (ECN). Particularly, decreased temporal correlation was observed in AN-r patients relative to healthy controls between the ECN functional connectivity maps and the anterior cingulate cortex (p < 0.05 corrected). Our results in AN adolescents may represent an early trait-related biomarker of the disease. Considering that the above mentioned network and its area are mainly involved in cognitive control and emotional processing, our findings could explain the impaired cognitive flexibility in relation to body image and appetite in AN patients.

Anorexia nervosa (AN) is a severe psychiatric disorder that typically tends to affect adolescent girls and young women, and its rate of mortality is the highest of all psychiatric disorders^1^. AN is characterized by typical psychiatric symptoms^2^: AN patients show an intense fear of weight gain or becoming fat, a distorted body image and food aversion, as well as clear physical signs as severe emaciation. In addition to an altered emotional focus on body and food^2^, AN patients show cognitive inflexibility and disturbances in social emotional functioning^3^,^4^, and it remains not well defined the role of psychiatric comorbidity in severity and outcome of AN in adolescents^5^ as in adults^6^. Furthermore, AN patients show higher levels of alexithymia and higher sensitivity to punishment compared to controls^7^,^8^. To date, AN aetiology is not fully understood, there is not an evidence-based treatment for AN, and the prognosis of AN remains poor^9^.

In the last decade, new functional and structural neuroimaging techniques were used to explore brain abnormalities in AN patients, trying to define the pathophysiology of AN. To date, the functional magnetic resonance (fMRI) is one of the most frequently used techniques on AN patients^9^. The majority of the fMRI studies have utilized specific stimuli, that were related with the main symptoms of AN as food-related, reward-related, and executive control-related tasks, as well as body-related tasks^9^,^10^. Overall, these fMRI studies showed neural functional alterations in each investigated symptomatological domain of AN. In particular, AN patients showed functional alteration in the cognitive control areas after investigating food image responses^11^,^12^, in the posterior parietal areas and prefrontal cortex-insula network after investigating the perceptive and affective components of body image distortion^10^,^13^, and in the ventral anterior cingulate-striato-thalamic loop after investigating cognitive-behavioural flexibility^14^. A current neurobiological model suggests that AN patients have an imbalance in information processing, linked to alterations of the ventral limbic and dorsal executive circuits^9^. In particular, the ventral limbic circuit (which comprises amygdala, anterior insula, anterior ventral striatum, anterior cingulate cortex, and the orbito-frontal cortex) and the dorsal executive-function circuit (which particularly includes dorsal regions of the caudate, dorso-lateral prefrontal cortex, and parietal cortex) are mainly involved in inhibitory decision making processes and reward-related behaviours and their alteration could sustain AN symptomatology^9^.

On the other hand, few studies have investigated resting state functional connectivity (RSFC) in AN patients. RSFC is an fMRI technique that allows mapping of temporal correlation between brain areas, based on spontaneous not-task related fluctuations of the BOLD signal in the resting brain^15^,^16^. Resting state networks (RSNs) are defined as the sets of brain areas that show a strong temporal coherence in the resting brain and are thought to represent specific frameworks of brain functional activity at rest^17^,^18^. Several networks have been identified and explored and, among these, the most studied network was the default-mode network^19^. Several RSFC studies have shown alterations in different psychiatric disorders, as obsessive-compulsive disorders^20^ and major depression^21^, and have been useful to shed more light in the pathophysiology of these disorders.

The first study that investigated RSFC in AN patients was conducted by Cowdrey and colleagues^22^. They studied a sample of adult recovered AN women, working on whole brain data driven analyses, and found increased RSFC between the default-mode network and the precuneus and the dorsolateral prefrontal cortex/inferior frontal gyrus in recovered AN patients compared to controls. Favaro and colleagues^23^, by means of a network of interest driven approach, found decreased activity in the ventral visual network in women with AN and those recovered from the disease compared with controls, and increased activity in the somatosensory network in those with AN. Similarly McFadden *et al.*^24^ showed reduced salience network activity in the anterior cingulate cortex (ACC) and reduced default mode network activity in the precuneus of AN patients as compared to controls. Also they found reduced activity of the sensory-motor network activity in the supplementary motor area and post-central gyrus of AN patients vs. controls and vs. recovered AN women. Furthermore, Boehm and colleagues^25^, also using a network of interest driven approach, found an increased functional connectivity between the frontal-parietal RSN and the left angular gyrus, and between the default-mode network and the left anterior insula/frontal operculum in a sample of unmedicated AN patients compared to controls. Two studies were conducted by applying a seed-based resting-state functional connectivity analysis on AN patients: a stronger synchronous activity between the dorsal ACC and retrosplenial cortex and between the dorsal ACC and precuneus was found in adult AN patients compared to controls^26^. On the other hand, Amianto and colleagues^27^ found alterations within the cerebellum network in AN and bulimia nervosa, as compared with controls. Recently, using degree centrality to investigate functional connectivity of the whole-brain network and then Granger causality to analyze effective connectivity, a reduced functional connectivity of the inferior frontal gyrus bilaterally and altered effective connectivity (i.e. from the right inferior frontal gyrus to the middle cingulate cortex, from the bilateral orbitofrontal gyrus to the right inferior frontal gyrus and from the bilateral insula to the left inferior frontal gyrus) were found in women with AN compared to controls^28^.

To date, only adult AN patients with long illness duration or adult recovered AN patients have been included in fMRI resting state studies. Starvation and malnutrition is associated with gray matter and white matter changes^29^ in AN and they can affect neuroimaging analyses^9^. Such limitations remain the main methodological question in the field of AN pathophysiology research and it remains to be established whether brain alterations of AN patients are the cause or the consequence of the disease and if specific brain functional alterations, as RSN alterations, may play a role in the pathophysiology of AN^9^. Studies on AN patients at the earliest stages of the disease may help to untangle this conundrum by limiting the confounding effects of long abnormal nutritional status of AN patients^30^.

Within this framework, our aim was to investigate RSFC in outpatient adolescents with AN in the earliest stages of the disease (i.e. AN in progress for less than 6 months at the time of scanning) compared to an age-matched control group, searching for abnormalities of RSNs. Considering that this is the first RSFC study in this stage of AN, we focused on the most widely studied RSNs^19^,^22^ to investigate whole brain functional connectivity, rather than only using a region of interest driven approach. Furthermore, we tested the relationship between resting state brain activity and *a priori* selected clinical variables related to main symptoms of AN (drive for thinness, body dissatisfaction, interoceptive awareness, perfectionism, bulimia, depression, trait anxiety, harm avoidance and body mass index).

## Results

### Demographic and clinical data

Table 1 shows the clinical features of the AN-restrictive type (AN-r) sample and the control group. The two groups had no differences in age. AN-r sample showed significantly lower BMI and significantly higher EDI-II subscales (i.e. Drive for thinness, Body dissatisfaction, Interoceptive awareness, and perfectionism), STAI-Trait, BDI, and Harm avoidance scores. Bulimia score was not significantly different between the two groups. AN-r patients did not meet the DSM-IV-TR criteria for other Axis I or Axis II disorders and they had no previous or current psychopharmacological treatments.

### VBM analysis and Resting State Functional Connectivity

Voxel-based morphometry (VBM) analysis, via SMP8 plus Diffeomorphic Anatomical Registration using Exponentiated Lie algebra (DARTEL) technique, showed no significant differences in gray matter (GM) between the AN group and the control group. The additional VBM analysis, via FSL, also showed no significant differences in GM between the AN group and the control group.

**Figure 1:**
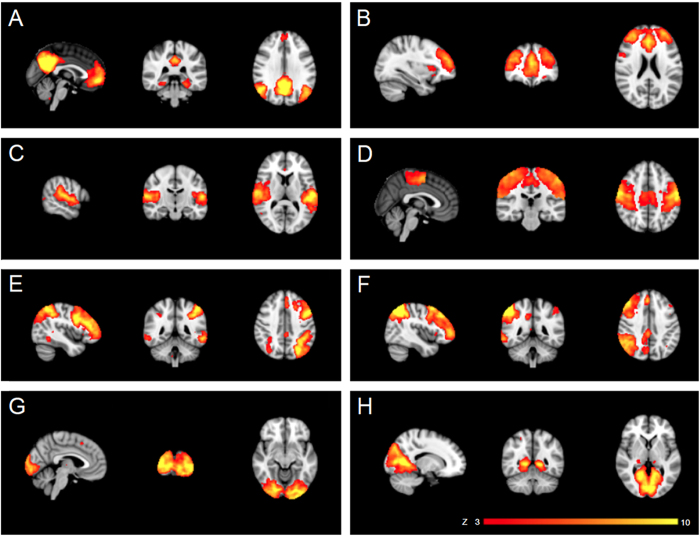
Resting-state networks (RSNs) identified as anatomically and functionally classical RSNs, used for the dual regression analysis. This figure shows sagittal, coronal and axial slices for the RSNs detected, overlaid onto the MNI152 standard brain. **A**, default mode network; **B**, executive control; **C**, auditory; **D**, sensory-motor; **E**, left fronto-parietal; **F**, right fronto-parietal; **G**, medial visual; **H**, lateral visual. RSNs are shown in FSL red-yellow color encoding using a 3 < ***z***-score < 10 threshold window.

**Figure 2:**
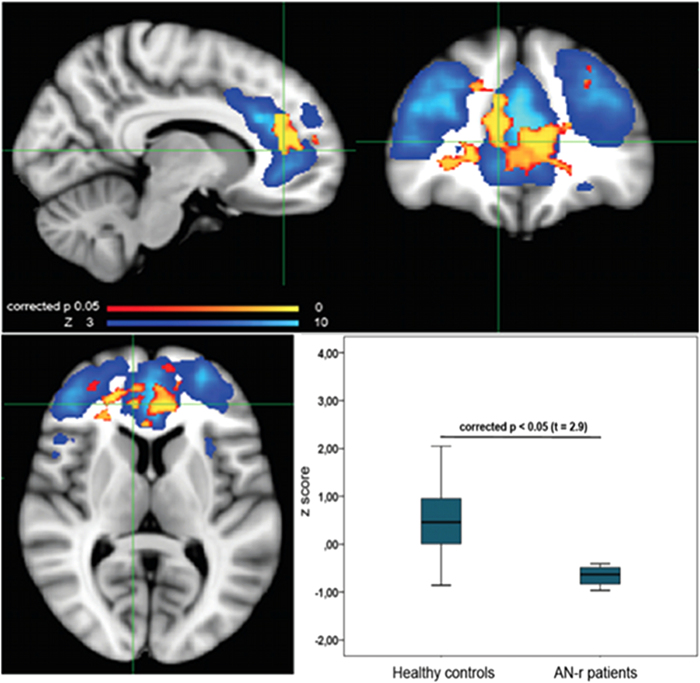
Significant decrease of resting state functional connectivity of ECN in AN-r patients compared to healthy controls, p < 0.05 FWE corrected, overlaid onto the ECN network (blue color scale) in the MNI152 standard brain. The box plot shows the difference of z score of AN-r patients compared to healthy controls within the ECN.

Independent component analysis (ICA) yielded 25 independent components representing group-averaged networks of brain regions with BOLD fMRI signals that were temporally correlated. Of these, 8 components were identified upon visual inspection as anatomically and functionally classical RSNs, previously reported in the resting-fMRI literature^19^: default mode network, executive control, auditory, sensory-motor, fronto-parietal (left and right), lateral visual and medial visual (Fig. 1). These networks corresponded to RSNs that have been described as highly stable over time^17^,^19^. The other components were discarded because they mainly reflected motion artefacts or BOLD signal drifts. The between group analysis of the voxelwise spatial distribution of the functional connectivity (FC) maps revealed significant differences in the executive control network (ECN). Significantly decreased RSFC (p < 0.05, FWE corrected) was observed in AN-r patients relative to healthy controls between the ECN functional connectivity maps and in a region of the ACC close to the border of the paracingulate gyri (t = 2.9, BA 9 (MNI coordinates: x = 10, y = 40, z = 8)) (Fig. 2). No differences in RSFC between the two groups were found in the FC maps of the other RSNs identified. The additional analysis that included GM maps as a covariate in GLM analyses of functional data confirmed significant differences in the executive control network (ECN) between groups in the voxelwise spatial distribution of the FC maps (see supplementary information and Fig. S1).

### Correlations between RSFC and clinical variables

**Figure 3:**
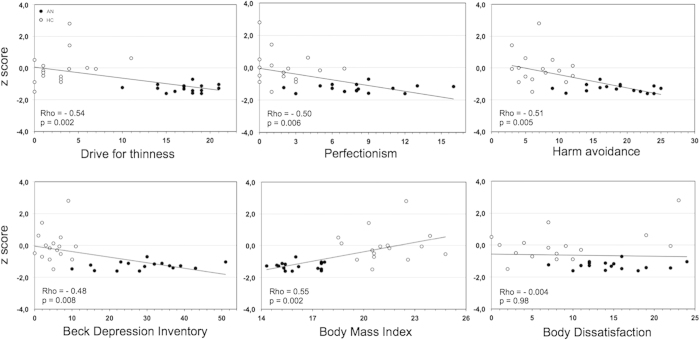
Graphs show the relationship between individual z scores within the ECN difference map and clinical variables in the AN-r patients (black dots) and healthy control subjects (white dots). Spearman’s Rho correlation coefficients and p values for data collapsed across all subjects are shown on the left lower corner of each panel.

Regional correlation analyses were conducted between a priori selected clinical variables of AN and the cluster maximal z-score of each participant within the mask of significant group-differences (i.e. ACC). Collapsing across subject group, the reduction of RSFC in the ECN network was negatively correlated with harm avoidance (Rho = −0.51; p = 0.005), drive for thinness (Rho = −0.54; p = 0.002), perfectionism (Rho = −0.50; p = 0.006), and BDI (Rho = −0.48; p = 0.008), and positively correlated with BMI (Rho = 0.55; p = 0.002) (Fig. 3). No significant correlations were found between the decreased RSFC and the other clinical scores assessed: body dissatisfaction (Rho = −0.004; p = 0.98); interoceptive awareness (Rho = −0.37; p = 0.05); Bulimia (Rho = −0.11; p = 0.58) and STAI-tr (Rho = −0.41; p = 0.03).

No significant correlations were found between the decreased RSFC and the *a priori* selected clinical variables in the AN-r sample and in the control group separately. Finally, the global correlation analyses showed no significant correlation between AN clinical variables and ECN maps in the AN-r sample.

## Discussion

The present study is the first work, to our knowledge, that investigates RSFC in adolescents with AN-r at the earliest stages of the disease (i.e. AN-r in progress for less than 6 months at the time of scanning). Our primary finding is that RSFC between Executive Control Network (ECN) and the ACC is decreased in AN-r adolescents compared to controls. The effect of AN-r on the ECN functional connectivity seems highly specific due to the lack of differences in the other RSNs.

Our primary results are partially consistent with the results of Lee *et al.*^26^ and McFadden *et al.*^24^ that found ACC functional connectivity alterations in AN patients compared to controls. The first research group, using a seed-based resting-state functional connectivity analysis, found a stronger synchronous activity between the dorsal ACC and retrosplenial cortex and between the dorsal ACC and precuneus in AN patients compared to controls^26^. McFadden *et al.*^24^ using fMRI signal during a conditioned stimulus task, showed that the ACC activity, as a part of the salience network, was reduced in women with AN compared to controls. In addition, they also showed that default mode network activity in the precuneus was reduced in women with AN-r compared to controls. On the other hand, the study of Cowdrey *et al.*^22^, investigating all RSNs, found only differences of the default mode network activity in a sample of recovered AN patients compared to controls. The differences between our results and the others could be due to the sample composition (e.g. six studies are based on data from adult samples and one study on data from a mixed sample of adult and adolescent patients)^22^,^23^,^24^,^25^,^26^,^27^,^28^. Particularly, AN patients with current or past long disease duration and several confounding factors (e.g. psychopharmacological treatment, psychiatric comorbidity) were included. Furthermore, some previous studies used different methodological approaches [i.e. selected networks and seed region analyses not including the ECN^23^,^25^,^27^ and a conditioned stimulus task respectively^24^]. In addiction, the study of Cowdrey *et al.*^22^ investigated on a sample of recovered AN patients: the approach to study recovered patients to find persistent or residual neural network alterations is eventually flawed by consequences and damage linked to disease duration, previous treatments and previous states of prolonged malnutrition. Overall, the fact that our sample is composed of adolescent AN-r patients at the earliest stages of the disease and with no confounding factors (i.e. psychiatric comorbidity, previous or current psychopharmacological treatment) may explain the differences between our results and the findings of the other studies. In particular, as GM abnormalities can affect functional analyses^31^, we suggest that the absence and the differences in direction changes (i.e. decrease vs increase) of the altered ACC connectivity could be also related to the grey matter vulnerability of ACC found in both long AN patients^32^ and patients recovered from AN^33^. Also, we could speculate that different RSNs are involved and/or affected depending on the different stage of AN in the recruited samples (e.g. earliest stages, long duration or recovery stage of AN).

Regarding the area of decreased RSFC, the ACC belongs to the limbic system and it is involved in cognitive, sensorimotor, and visceral functions, as well as in emotion processing (e.g. unpleasantness)^34^. Particularly, its subregions (i.e. subgenual region and pregenual region) store negatively valenced memories and are engaged in positively valenced events respectively^34^. As it regards intrinsic RSNs, the ACC is considered a part of the ECN^19^. This network also covers several medial–frontal areas, including the paracingulate cortex and plays a key role in several cognition paradigms, action–inhibition, emotion, and perception–somesthesis–pain^19^. On the other hand, the fMRI studies that have assessed intrinsic network activity across task performance pointed out that the ACC shows multiple intrinsic connectivities^35^,^36^,^37^. Several fMRI studies have highlighted two distinct networks: the salience network and the ECN^37^. The first covers the dorsal ACC and orbital frontoinsular cortices and it is correlated with anxiety but not executive function. The second covers dorsolateral frontal and parietal neocortices and it is correlated with executive function^37^. Overall, the ACC is a key area of the brain, shows multiple functions and multiple intrinsic connectivities at rest, and seems to be involved in both cognitive and affective control processes^36^.

Secondly, we found that, after collapsing data across subject groups, the decrease of RSFC in the ACC correlated negatively with drive for thinness, perfectionism, harm avoidance and depression scores while it correlated positively with BMI. This preliminary evidence seems to link the early ACC functional connectivity alterations to the core symptoms and traits of AN. Concerning these results, it is noteworthy that drive for thinness, perfectionism and harm avoidance are described as childhood predisposing factors that precede the onset of an eating disorder^38^,^39^. Furthermore, the ACC has been suggested to be specifically involved in body image distortion^40^, impaired cognitive-behavioural flexibility^14^, excessive cognitive control of appetite^41^, and perfectionism^42^ in AN patients.

Overall, our results lead us to propose two interpretations. The first is that the RSFC alterations of the ACC may be due to malnutrition and weight loss in AN. The positive correlation between the ACC alterations and BMI, found after collapsing data across subject groups, seems to support this interpretation and could lead to suggest that ACC abnormalities are related to malnutrition and weight loss. However, no significant differences were found in gray matter volumes between AN-r patients and controls and the additional functional analysis using gray matter maps as covariate (to assess the possible role of potential sub-threshold differences of gray matter) confirmed the ACC alterations. Thus, it can be assumed that the functional connectivity alteration is not related to gray matter loss or, at least, that malnutrition and weight loss have not yet produced effects on brain structure. On this regard, there are no data, to our knowledge, that show a specific functional vulnerability of ACC to starvation or weight loss. In addition, no gray matter decrease of the ACC has been found in adolescents at the early stages of AN^30^,^43^.

The second interpretation is that the resting-state functional connectivity alterations of the ACC are somehow directly related to the earliest stages of AN. On this perspective such altered resting-state functional connectivity could be proposed as an early neural biomarker of AN. This hypothesis seems to be supported by the fact that ACC, as part of the ECN, is involved in cognitive control, as well as in emotional processing. Furthermore in our study, the RSFC alterations of the ACC were negatively correlated with drive for thinness, perfectionism and harm avoidance scores. Thus, we may suggest that the early RSFC alterations of the ACC may be involved in the pathophysiology of AN. Particularly, the ACC functional alterations could support the impaired cognitive flexibility and ruminative preoccupation on body image and weight of AN patients, leading to the characteristic behavioural vicious circle of AN. Furthermore, given the association of ACC activation with conflicts in information processing requiring response override^44^,^45^, its alteration may explain the altered capacity of AN patients in the processing and integration of bodily perceptions^46^,^47^.

The present study has some methodological advantages, as well as several limitations that should be taken into consideration. The main strength of the present study is the homogeneity of the sample. The clinical sample is composed of AN-r adolescent patients at the earliest stages of the disease (i.e. AN-r in progress for less than 6 months at the time of scanning) and without confounding conditions (i.e. no psychiatric comorbidity, no previous or current psychopharmacological treatment). Considering that brain atrophy can affect functional connectivity analysis^31^, the absence of significant differences between the two groups in gray matter volumes is a further strength of the study. However, we cannot exclude the possibility that our results are partially related to brain reorganization at the earliest stages of AN-r (e.g. functional resilience in response to weight loss and starvation). The main limitation of the current study is its cross-sectional design. However, our purpose was to investigate a homogeneous sample of AN-r patients whose disease had been in progress for a very limited time period in order to highlight RSFC alterations at the earliest stages of AN-r. The second limitation is the small sample size: this was due to the strict inclusion and exclusion criteria as well as to the low incidence of the disorder.

In conclusion, the present study showed that our sample of adolescent patients with AN-r at the earliest stages of the disease had significant RSFC decrease between the ECN network and the ACC. The early functional connectivity alterations between the ECN and the ACC, which are also involved in the cognitive control and emotional processing, could have a role in the pathophysiology of AN and could explain the impaired cognitive flexibility in relation to body image and appetite in AN patients. We believe that controlled longitudinal studies are needed to confirm our findings and to identify the role of such functional modifications. Exploration of RSFC in AN, also analysing topological network properties (e.g. modularity, hub-ness, small-worldness), might be used to assess brain functional plasticity induced by therapeutic strategies aimed at improving information processing and cognitive flexibility in the earliest stages of AN.

## Methods

### Participants

Overall, 34 adolescents (18 outpatients with AN-r and 16 healthy subjects) were recruited and scanned. The study sample was composed of 16 adolescent females with AN-r and 16 age-matched healthy adolescent females. Two AN-r patients were excluded due to MRI artefacts that significantly decreased the image quality, secondary to orthodontic braces. The AN-r subjects were recruited in a non-profit outpatient treatment centre for eating disorders (i.e. “La cura del girasole ONLUS”) in Rome (Italy) and met the DSM-IV-TR^2^ diagnostic criteria for AN-r. The inclusion criteria for the clinical sample were: a diagnosis of AN-r in accordance with DSM-IV-TR criteria^2^; 13–18 years of age; duration of AN-r less than 6 months at the time of scanning; right-handedness. The exclusion criteria were a previous history of other eating disorders; the presence of a binge eating/purging type of AN; a current or previous psychopharmacological treatment; the presence of other current or previous psychiatric disorders (DSM-IV-TR); history of neurological diseases or head trauma; concomitant medical diseases; and the presence of any absolute contraindication for MRI. All patients were under diagnostic evaluation for AN (all procedures of the study were completed within 1 week after the first clinical interview).

The control sample was recruited in a high school of the same geographic area of the patients sample to obtain age and socio-economic features similar to the clinical sample. The inclusion criteria for the control group were: 13–18 years of age and right-handedness. The exclusion criteria were previous or current eating disorders; previous or current other psychiatric disorders (DSM-IV-TR); previous or current psychopharmacological treatment; history of neurological problems or head trauma; concomitant medical diseases; and the presence of any absolute contraindication for MRI.

At the time of scanning, all participants of both groups had received similar schooling [AN group: mean (years) = 9.4 ± 1.09; control group: mean (years) = 9.8 ± 1.4. t = −.730, p = 0.47] and had no specific training or skills^48^.

The present study was conducted according to the declaration of Helsinki and it was approved by the “La cura del girasole” ONLUS institutional review board. Written informed consent was directly obtained from the parents of those participants younger than 18 and from those participants who had reached the age of 18.

### Clinical assessment and tools

The same psychopathological assessment was used for all participants. Particularly, diagnosis of AN and other current or past EDs was made by a clinical interview that was performed according to the eating disorders section of the Structured Clinical Interview for DSM-IV^49^. Diagnosis of past or current other Axis I disorders was made in accordance with DSM-IV-TR criteria^2^ by a comprehensive clinical interview. The Italian version of the Structured Clinical Interview for Axis II Disorders (SCID II)^50^ was used to assess personality disorders in patients older than 16 years of age. In addition, all participants also completed the Italian version of the Eating Disorder Inventory (EDI-II)^51^ for drive for thinness, body dissatisfaction, interoceptive awareness, perfectionism and bulimia; the Italian version of the Beck Depression Inventory-II (BDI-II)^52^; the Italian version of the State-Trait Anxiety Inventory (STAI-Y)^53^ for trait anxiety; and harm avoidance was assessed using the temperament and character inventory^54^.

The EDI-2 is a self-report questionnaire that is used to measure disordered eating behaviours and attitudes and personality traits common to individuals with ED. The BDI is a self-report questionnaire that is used to assess the presence of depressive symptomatology. The STAI is a self-report questionnaire that is used to assess both trait anxiety (STAI-trait) and the level of anxiety at the time of the evaluation (STAI-state). The TCI is a 240-item self-administered questionnaire divided into 7 dimensions. In our study we only used one of the temperament dimensions: Harm avoidance. All interviews and self-report questionnaires were carried out by the first author with 10 years expertise in eating disorders in children and adolescents and in child and adolescent psychiatry and who was specifically trained to use the diagnostic tools applied.

### fMRI Data Acquisition

Imaging data were acquired using a Siemens 1.5-T MAGNETOM Avanto (Siemens, Erlangen, Germany) whole body scanner equipped with a 12-element designed Head Matrix coil, as part of the standard system configuration. Resting state fMRI data were acquired after a 3.55 minutes morphological scan, used to improve registration of BOLD-weighted images and for anatomical segmentation of gray matter. A 3D-MPRAGE T1 weighted sequence was conducted: TR = 1900 ms, TE = 3.37 ms, TI = 1100 ms, flip angle = 15°, FOV = 256 mm × 192 mm, NEX = 1, matrix = 256 × 192, 1.00 × 1.00 mm^2^ in-plane resolution, horizontal slices with slice thickness of 1.3 mm and no gap.

Whole brain functional scans were acquired in 25 contiguous axial slices approximately parallel to the anterior-posterior commissure plane with interleaved multi-slice T2* echo-planar imaging according to the following parameters: TR = 3.56 s, TE = 50 ms, field of view = 22 cm, flip angle = 90°, voxel size = 3.4 × 3.4 × 3 mm, slice thickness = 3 mm, no inter-slice gap. For each participant, a total of 80 volumes during 4.50 min were acquired. fMRI scanning was carried out in darkness, and the participants were explicitly instructed to relax with their eyes closed, think of nothing in particular, not to fall asleep and stay as much still as possible. At the end of the MR examination, all participants were asked about their feelings during the scan and the tendency to sleep during the scanning. None of the subjects fell asleep or reported significant feelings during the scan.

Structural T1-weighetd MR data was analysed using SPM version 8 (SPM8; Wellcome Department of Imaging Neuroscience, London, United Kingdom) plus DARTEL technique^55^. Structural data corrected for image-intensity non uniformity were segmented into GM, WM, and CSF by a unified tissue-segmentation procedure. Segmented GM and WM images were spatially normalized to the customized template in the standardized anatomic space by using DARTEL^55^ at a resolution of 2 × 2 × 2 mm3. Then, normalized images were modulated using the Jacobian determinants derived from previous DARTEL spatial normalization, to preserve GM and WM native volumes. Finally, the modulated images were smoothed using an 8-mm FWHM Gaussian kernel. Differences in GM volume between the patient group and control group were assessed by a 2-sample t-test in SPM8. Group comparisons were assessed using the false discovery rate at a threshold of p < 0.05, corrected for multiple comparisons. An additional VBM analysis was performed using FSL tools^56^ to replicate previous resting state studies on AN patients^22^,^23^. In brief, structural data was brain-extracted using BET tool^57^ in order to remove skin and skull tissues. A tissue-type segmentation was performed using FAST^58^ package, producing GM, white matter (WM) and cerebral spinal fluid (CSF) volumes. The GM (partial volume) images were then aligned to MNI152 standard space using the affine registration tool FLIRT,^59^,^60^ followed by nonlinear registration using FNIRT^61^. Then, the registered images were averaged to create a study-specific template in order to reduce the effect of inter-subject brain variability during the registration procedure, to which the native GM images were non-linearly re-registered. The resulting images were modulated (to correct for local expansion or contraction) and smoothed with an isotropic Gaussian kernel with a sigma of 4 mm (corresponding to a full width at half maximum of 9.4 mm) for the TFCE-based analysis^62^. Finally, differences in GM volume between the patient group and control group were assessed by voxelwise GLM, applied using permutation-based non-parametric testing (10,000 permutations)^63^ with a statistical threshold value of p < 0.05, corrected for multiple comparisons.

### Data Processing and Statistics

Resting-state functional connectivity analysis was carried out using the independent component analysis (ICA) tool MELODIC (Multivariate Exploratory Linear Optimized Decomposition into Independent Components)^64^, Version 3.13 part of FSL v. 5.0.4 (FMRIB’s Software Library http://fsl.fmrib.ox.ac.uk/fsl). Single-subject pre-processing was carried out using FEAT (FMRI Expert Analysis Tool), Version 6.00, part of FSL v. 5.0.4 (FMRIB’s Software Library http://fsl.fmrib.ox.ac.uk/fsl). Pre-statistical processing consisted of motion correction using MCFLIRT^60^, brain extraction using BET and spatial smoothing using a Gaussian kernel of full-width at half-maximum of 5 mm^57^. Gross signal drifts (due to scanner instabilities or systemic physiological fluctuations) were attenuated by applying a high-pass filtering cut-off, set at 150 seconds (0.007 Hz)^22^. Registration to high resolution structural and/or standard space images was carried out using FLIRT^59^,^60^. EPI volumes were registered to the individual’s structural scan using FLIRT_BBR (Boundary-Based Registration) tool^65^. Registration from high resolution structural to standard space was then further refined using FNIRT nonlinear registration^66^,^67^. To carry out group-wise ICA, a single 4D data set was created by temporally concatenating preprocessed functional data, containing 80 time points for each subject. Dimensionality of Group-ICA was carried out at different numbers of components (i.e. 20, 25, 30, 35, 40)^19^,^64^,^68^. Finally, 25 components were used as explained data variance was sufficient to obtain good estimates of the signals and well known RSNs were identified^19^. RSNs of interest covered the entire brain and were selected by visual inspection against sets of previously defined maps^19^,^64^. The set of spatial maps from the group-average analysis was used to generate subject-specific versions of the spatial maps, and associated time series using dual regression technique^69^,^70^. First, for each subject, the group-average set of spatial maps is regressed (as spatial regressors in a multiple regression) into the subject’s 4D space-time dataset. This results in a set of subject-specific time series, one per group-level spatial map. Next, those time series are regressed (as temporal regressors, again in a multiple regression) into the same 4D dataset, resulting in a set of subject-specific spatial maps, one per group-level spatial map, which are then tested voxelwise for statistically significant differences between the groups using FSL’s *randomise* permutation-testing tool (10,000 permutations)^63^. Clusters were determined by using threshold-free cluster enhancement (TFCE)^62^ and a family-wise error (FWE) corrected cluster significance threshold of p < 0.05.

Since data from voxel–based morphometry studies have shown global and regional decreases of gray matter volume in AN^29^, and type I and II errors are reduced by the inclusion of voxelwise tissue information as a covariate in GLM analyses of functional data^31^, especially when structural differences are present, we conducted VBM analysis in our sample. Due to the lack of significant differences in GM volumes between the patient group and the control group, we did not include GM as a nuisance variable in GLM analyses of functional data. As additional analysis, to investigate whether functional differences survive potential sub-threshold differences of gray matter, we included voxelwise tissue information (i.e. GM maps of FSL VBM) as a covariate in GLM analyses of functional data^31^.

Anatomical localization of significant clusters was established according to the Harvard-Oxford Cortical Structural Atlas included in the FSL (http://www.fmrib.ox.ac.uk/fsl/data/atlas descriptions.html).

Demographic and clinical data were tested for normal distribution using the Kolmogorov-Smirnov test. The Student’s t test was used for normally distributed variables and the Mann-Whitney U test for non-normally distributed variables. The relationship between RSFC regional (cluster maximal z-score) and global (FC maps) differences and clinical variables (drive for thinness, body dissatisfaction, interoceptive awareness, perfectionism, bulimia, depression, trait anxiety, harm avoidance, and BMI) were tested by non parametric Spearman’s correlation analyses at the significant level of p < 0.01 and using GLM analysis in FSL at significant level of p < 0.05 corrected, respectively.

## Additional Information

**How to cite this article**: Gaudio, S. *et al.* Altered resting state functional connectivity of anterior cingulate cortex in drug naïve adolescents at the earliest stages of anorexia nervosa. *Sci. Rep.* **5**, 10818; doi: 10.1038/srep10818 (2015).

## Supplementary Material

Supplementary Information

## Figures and Tables

**Table 1:** Clinical features of participants.

	**AN-r (N = 16)**		**Controls (N = 16)**			
	**Mean**	**(SD)**	**Mean**	**(SD)**	**Statistic**^**1**^	***p***
Age (years)	15.8	(1.7)	16.3	(1.4)	1.00	.33
BMI	16.2	(1.2)	21.1	(1.9)	-8.897	<.001
Age of onset of AN (years)	15.4	(1.6)	–	–	–	–
Duration of AN (months)	4.0	(1.8)	–	–	–	–
Lifetime lowest BMI	16.1	(1.2)	–	–	–	–
Drive for thinness^2^	17.3	(2.9)	2.8	(3.1)	13.626	<.001
Body dissatisfaction^2^	14.9	(4.5)	8.2	(7.3)	3.104	.004
Interoceptive awareness^2^	10.9	(6.4)	2.3	(3.3)	4.807	<.001
Perfectionism^2^	8.7	(4.0)	1.9	(2.1)	6.090	<.001
Bulimia^2^	2.4	(2.2)	1.3	(1.8)	1.659	.11
STAI – Trait anxiety	53.8	(14.1)	29.8	(5.2)	6.414	<.001
BDI-II	30.3	(11.5)	5.1	(3.2)	8.467	<.001
Harm avoidance^3^	19.1	(4.5)	6.9	(3.3)	7.687	<.001

Note. AN-r = Anorexia Nervosa-restrictive type. N = Numbers. SD = Standard deviation. BMI = body mass index. STAI = State trait anxiety inventory. BDI-II = Beck depression inventory. ^1^Student’s T Test. ^2^Subscales of Eating disorders inventory (EDI-II). ^3^Subscale of temperament and character inventory.
