# Supplementary material for: Altered resting state functional connectivity of anterior cingulate cortex in drug naïve adolescents at the earliest stages of anorexia nervosa
Source: Sci Rep. 2015 Jun 4;5:10818. doi: 10.1038/srep10818 (PMC4455287; doi:10.1038/srep10818)
Supplement: Supplementary Information [file srep10818-s1.pdf]

Altered resting state functional connectivity of anterior cingulate cortex in drug naïve adolescents at the earliest stages of anorexia nervosa

Santino Gaudio, Claudia Piervincenzi, Bruno Beomonte Zobel, Francesca Romana Montecchi, Giuseppe Riva, Filippo Carducci, Carlo Cosimo Quattrocchi

Supplementary Results

Additional analyses of Resting State Functional Connectivity

The additional analysis with GM maps as a covariate in GLM analyses of functional data showed that the between group analysis in the voxelwise spatial distribution of the functional connectivity (FC) maps confirms significant differences in the executive control network (ECN).

Significantly decreased RSFC ($p < 0.05$, FWE corrected) was observed in AN-r patients compared to healthy controls between the ECN functional connectivity maps and in a region of the ACC close to the border of the paracingulate gyri ($t = 3.16$, BA 9 (MNI coordinates: x=10, y=42, z=6) (Figure S1).

Supplementary Figure S1:

Clusters of significant decrease in resting state functional connectivity of ECN in AN-r patients compared to healthy controls using GM maps as covariate in GLM analysis ($p < 0.05$ FWE corrected) are overlaid onto the ECN network (blue color scale) in the MNI152 standard brain. The maximal peak (MNI coordinates: x=10, y=42, z=6; $t = 3.16$) corresponds to a region of the anterior cingulate cortex close to the border of the paracingulate gyri, thus it is consistent with the results of Figure 2.

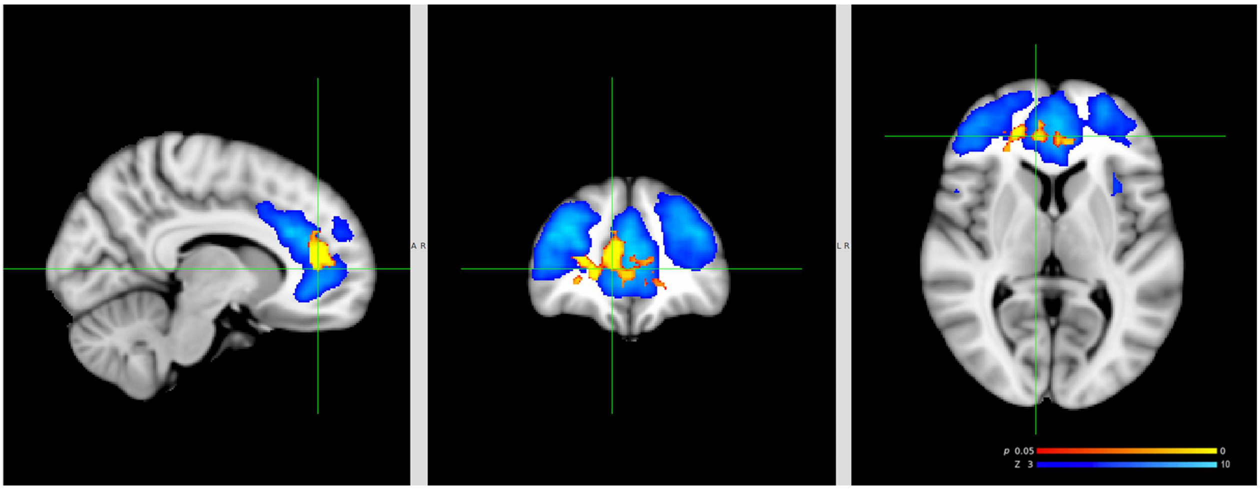
A R
L R
p 0.05
0
Z 3
10
